# Antibiotic-Induced Primary Biles Inhibit SARS-CoV-2 Endoribonuclease Nsp15 Activity in Mouse Gut

**DOI:** 10.3389/fcimb.2022.896504

**Published:** 2022-07-28

**Authors:** Yao Ma, Mei Luo, Yusheng Deng, Xiaoman Yang, Xionglue Wang, Guozhong Chen, Zixin Qin, Yun Deng, Meiling Nan, Yang Chen, Peihui Wang, Hong Wei, Lijuan Han, Xiaodong Fang, Zhi Liu

**Affiliations:** ^1^ Department of Biotechnology, Key Laboratory of Molecular Biophysics of the Ministry of Education, College of Life Science and Technology, Huazhong University of Science and Technology, Wuhan, China; ^2^ Department of Scientific Research, KMHD, Shenzhen, China; ^3^ Key Laboratory for Experimental Teratology of Ministry of Education and Advanced Medical Research Institute, Cheeloo College of Medicine, Shandong University, Jinan, China; ^4^ State Key Laboratory of Dampness Syndrome of Chinese Medicine, The Second Affiliated Hospital of Guangzhou University of Chinese Medicine, Guangzhou, China; ^5^ State Key Laboratory of Agricultural Microbiology, College of Animal Sciences and Technology, Huazhong Agricultural University, Wuhan, China

**Keywords:** SARS-CoV-2, Nsp15, antibiotics, gut microbiota, primary bile acids

## Abstract

The gut microbiome profile of COVID-19 patients was found to correlate with a viral load of SARS-CoV-2, COVID-19 severity, and dysfunctional immune responses, suggesting that gut microbiota may be involved in anti-infection. In order to investigate the role of gut microbiota in anti-infection against SARS-CoV-2, we established a high-throughput *in vitro* screening system for COVID-19 therapeutics by targeting the endoribonuclease (Nsp15). We also evaluated the activity inhibition of the target by substances of intestinal origin, using a mouse model in an attempt to explore the interactions between gut microbiota and SARS-CoV-2. The results unexpectedly revealed that antibiotic treatment induced the appearance of substances with Nsp15 activity inhibition in the intestine of mice. Comprehensive analysis based on functional profiling of the fecal metagenomes and endoribonuclease assay of antibiotic-enriched bacteria and metabolites demonstrated that the Nsp15 inhibitors were the primary bile acids that accumulated in the gut as a result of antibiotic-induced deficiency of bile acid metabolizing microbes. This study provides a new perspective on the development of COVID-19 therapeutics using primary bile acids.

## Background

To date, the coronavirus disease (COVID-19) pandemic has infected over 500 million people and resulted in more than six million deaths worldwide (WHO, 2022b). Coronavirus SARS-CoV-2 is the causative agent of COVID-19 with limited specific medicine or treatment. A large number of antiviral agents have been evaluated for SARS-CoV-2 since the breakout of COVID-19 (Izda et al., 2021). However, remdesivir is the only antiviral recommended for COVID-19 treatment in Europe, with efficacy and safety recently questioned (Scavone et al., 2022). The World Health Organization (WHO) reported that only 57 countries have vaccinated over 70% of their population by May 22, 2022, almost all of which are high-income countries. The role of antiviral agents in COVID-19 treatment is still being investigated.

Apart from the common respiratory symptoms, a number of COVID-19 patients showed gastrointestinal symptoms, such as diarrhea (2.0%–55.0%), nausea (1.0%–27.5%), vomiting (1.0%–12.5%), lack of appetite (10.1%–39.7), and abdominal pain (0.98%–5.8%) (Zhang et al., 2021). A study exploring the association between fecal microbiome composition and SARS-CoV-2 transcriptional activity has showed that the patients with active SARS-CoV-2 gastrointestinal tract (GI) infection harbor more opportunistic pathogens and decreased abundance of short-chain fatty acid (SCFA) producing bacteria (Zuo et al., 2021a). Moreover, the gut microbiome profile of COVID-19 patients has been found to be consistent with COVID-19 severity and dysfunctional immune responses, featuring the depletion of *Faecalibacterium prausnitzii*, *Eubacterium rectale*, and *Bifidobacteria* which have been identified with immunomodulatory potential (Yeoh et al., 2021). Taken together, gut microbes may serve as indicators of SARS-CoV-2 infection and may be involved in anti-infection. Therefore, it is important to examine and screen intestinal resources for substances that may be applied in COVID-19 therapy.

The reported targets of anti-coronavirus therapies focus on viral mRNA synthesis and replication, among which, a common target functioning in mRNA synthesis is the coronavirus endoribonuclease (EndoU). EndoU is encoded by nonstructural protein 15 (*nsp15*), a specific marker for vertebrate nidoviruses, which is involved in viral mRNA production and transcription (Deng and Baker, 2018). The conserved aspartate residue in the coronavirus Nsp15 protein (Asp-298 of Coronavirus humano 229E (HCoV-229E) and its homologous site) is essential for its correct folding and RNA synthesis capacity (Ivanov et al., 2004). In addition to its involvement in regulating viral replication, the Nsp15 protein is also critical in evading host dsRNA sensors in macrophages (Deng et al., 2017). The Nsp15 protein of SARS-CoV exists mainly as a hexamer that cleaves at 3’ of uridylates of RNAs, and preferentially cleaves at the U30 site of the highly conserved RNA element, stem-loop II motif (s2m), and in the 3’ non-translated region (UTR) of the virus genome (Bhardwaj et al., 2006). Since the s2m RNA element is highly conserved in nidoviruses and is not present in the human genome, Nsp15 is considered an important target for antiviral drug development (Robertson et al., 2005; Canal et al., 2021). As for Nsp15, crystal structure determination and endoribonuclease activity revealed that the active site residues of SARS-CoV-2 are conserved to that of SARS-CoV, both in terms of sequence and conformation (Kim et al., 2020). Accordingly, in this study, we chose Nsp15 as a screening target for COVID-19 therapeutics. And we successfully established high-throughput *in vitro* screening system of Nsp15.

Traditional Chinese patent medicines (CPMs) have been widely used in the clinical treatment of COVID-19 in China, and some of these CPMs have been shown to have anti-viral effects (Zhuang et al., 2020; Huang et al., 2021). In China, three traditional Chinese medicine (TCM) decoctions and three formulated Chinese medicines (as six TCM recipes) have been proven to be the most effective TCMs in treating COVID-19 patients (Luo et al., 2020). Among the six TCM recipes, Jinhua Qinggan granules (JHQGG) have been shown to significantly increase the 7-day viral clearance rate and shorten recovery time from pneumonia (Liu et al., 2020), while Lianhua Qingwen capsules (LHQWC), one of six TCM recipes combined with anti-influenza drugs, more effectively alleviated clinical symptoms and improved treatment efficacy in patients with mild COVID-19 (Huang et al., 2021). A variety of TCM herbal components have been reported to influence the abundance and diversity of gut microbiota, which in turn, affect the efficacy of TCM (Lin et al., 2020). Particularly, TCM ingredients can be transformed by gut microbiota into small chemical molecules to achieve the pharmacological functions. For example, gut microbiota can convert ginsenosides in orally administrated ginseng to compound K, which shows higher anti-tumor, anti-inflammatory, and anti-allergic activity than ginsenoside Rb1 (Wang et al., 2011; Kim et al., 2013). Thus, we evaluated the activity inhibition on the target by substances of intestinal origin using a mouse model and attempted to explore the interaction between gut microbiota and clinical traditional CPMs. The results unexpectedly revealed that it is antibiotic treatment, not CPMs, which induced the appearance of substances with Nsp15 activity inhibition in the intestine of mice. Further analysis revealed that antibiotic treatment led to the depletion of flora functioning in bile acid metabolism, which resulted in the accumulation of primary bile acids and displayed an inhibitory effect on Nsp15 in the gut.

## Results

### Establishment of an *in vitro* System for Screening Nsp15 Inhibitors

**Figure 1:**
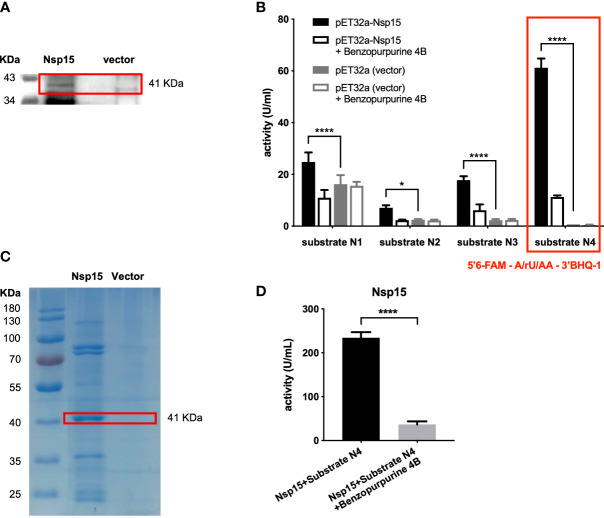
Establishment of *in vitro* screening system for Nsp15 inhibitors. **(A)** Nsp15 expression was detected by Western blotting. *E. coli* BL21(DE3) cells containing pET32a plasmids with or without (vector) recombinant SARS-CoV-2 Nsp15 were grown to OD_600_ of 0.9 - 1.0 and then induced with 1 mM IPTG. After additional incubation at 18°C for 16 h, cells in 1 ml of bacterial culture were collected for Western blotting with anti-His-6 antiserum. **(B)** Analysis and comparison of Nsp15 substrates using endoribonuclease assay. Reactions were performed in the reaction buffer containing 50 mM Tris-HCI pH 7.5, 100 mM KCI, 5 mM MnCl_2_, and 1 mM DTT, at 30°C. Benzopurpurine 4B is an inhibitor of SARS-CoV Nsp15. Enhanced fluorescence due to the cleavage of the fluorogence substrate RNA was monitored at 518 nm with excitation at 492 nm by a fluorescence plate reader. Values expressed are means ± S.D. from three independent experiments. Significance was determined separately for each substrate by One-way ANOVA; *p*-value: *, < 0.05, ****, < 0.0001. **(C)** Purified Nsp15 were detected by Coomassie staining. His-tagged Nsp15 proteins were purified by Ni-NTA Agarose and separated by SDS-PAGE followed by Coomassie staining; we spliced the ladder next to the destination protein band. **(D)** Endoribonuclease assay of purified Nsp15. The endoribonuclease activity of purified Nsp15 was determined by its ability in cleaving fluorogenic RNA substrate N4. Values expressed are means ± S.D. from three independent experiments. Significance was determined by *t*-test; *p*-value: ****, < 0.0001.

The recombinant Nsp15, with expected molecular weight of 41 KDa, was successfully expressed in *E. coli* BL21 (DE3) (**Figure 1A**). Then, we developed the enzymatic activity detection system for Nsp15 based on the fluorescence resonance energy transfer (FRET) mechanism (Boute et al., 2002). Benzopurpurin B was chosen as a Nsp15 inhibitor control according to a study of SARS-CoV (Ortiz-Alcantara et al. 2010). Four fluorogenic RNA substrate candidates were assessed in the construction of the Nsp15 endoribonuclease screening system, based on the characteristic that Nsp15 preferentially cleaves at uridylate bases that are not involved in base-pairing (Bhardwaj et al., 2006). The substrates were 5’6-FAM-GAAGCGAAACCC/rU/AAG-3’BHQ1 (substrate N1), 5’6-FAM-GCGGAGCACGA/rU/CGAG-3’BHQ1 (substrate N2), 5’6-FAM-UACGAUCG-3’BHQ1 (substrate N3), and 5’6-FAM-A/rU/AA-3’BHQ1 (substrate N4). Among them, substrate N4 exhibited the highest enzymatic activity, the minimal non-specific degradation (**Figure 1B**), thus was selected for subsequent high-throughput screening. The Nsp15 purified with Ni-NTA Agarose exhibited high activity on cleaving fluorogenic RNA substrate N4 (**Figures 1C, D**). We further utilized the crude enzyme Nsp15 for the high-throughput screening due to the low consumption.

At this point, the Nsp15 endoribonuclease screening system *in vitro* was successfully established.

### Antibiotic Treatment Induces the Production of Nsp15 Inhibitors in the Gut

Traditional Chinese patent medicines (CPMs) have been widely used in the clinical treatment of COVID-19 in China, and have a significant role in the treatment, according to a number of clinical reports (Zhuang et al., 2020). Conversely, clinical data and experiments on the macaque model of COVID-19 have suggested that COVID-19 infection reduces the diversity of intestinal microbiome, further affecting the function of the gut microbiome (decreased SCFA synthesis, impaired conversion of primary to secondary bile acids, upregulation of tryptophan metabolism) and promoting intestinal inflammation, which lead to the disruption of gut immune homeostasis (Zuo et al., 2021b). Therefore, we investigated whether CPMs function through intestinal bacteria, using LHQWC (Tao et al., 2013; Ding et al., 2017) and JHQGG (Liu et al., 2020) as examples, since their pharmacological effects are anti-viral (Zhuang et al., 2020) and are recommended for clinical treatment of COVID-19 (Wei, 2020).

**Figure 2:**
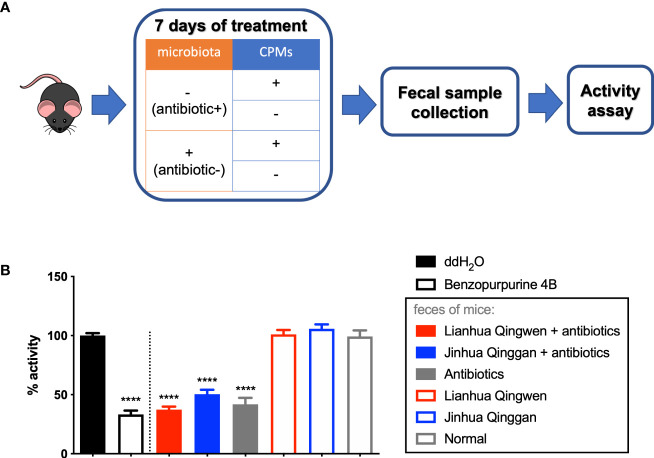
Antibiotic-induced alteration of gut microbiome led to inhibitory effect on Nsp15 activity. **(A)** Illustration of animal assay. C57BL/6J males were supplied with or without antibiotic cocktail for 7 days to illustrate mouse model with or without gut microbiota. These mice were administrated with or without CPMs at the same time in order to examine the interaction between CPMs and microbiota, by assessing the impact of fecal samples on SARS-CoV-2 Nsp15 activity. Fecal pellets of each mouse were collected at the end of experiment, and supernatant of fecal homogenate in ddH_2_O was subjected to activity assay. **(B)** Analysis of the effect of fecal samples on Nsp15 activity. The reaction systems with ddH_2_O and Benzopurpurine 4B instead of the sample to be tested were negative and positive controls, respectively. Relative activity (% of activity) was calculated for each sample by comparing with the negative control. Values expressed are means ± S.D. from three independent experiments for no less than three biological replicates. Significance was determined by One-way ANOVA; *p*-value: ****, < 0.0001.

By using antibiotic treatment, followed with gavage of CPMs in the mice, we obtained mouse fecal samples with CPM treatment and microbiome depletion (**Figure 2A**). Similarly, the samples of mice treated with or without CPMs and with or without intestinal microorganisms were obtained. These samples were then subjected to *in vitro* assay for the inhibitory effect on Nsp15. As a result, fecal samples from the mice treated with the CPMs alone could not inhibit Nsp15 (**Figure 2B**). Surprisingly, the fecal samples of antibiotic-treated mice, irrespective of CPM treatment, inhibited Nsp15 enzyme activity (**Figure 2B**). These data lead to the conclusion that CPMs do not inhibit the tested targets *via* gut microbes, while antibiotic treatment induces the production of Nsp15 inhibitors in the mouse intestine.

### Compositional Changes of Gut Microbiome Contribute to the Emergence of Nsp15 Inhibitors

Antibiotic treatment, along with possible residual antibiotics, has been reported to alter gut microbiome and induce changes in metabolic homeostasis in mice (Zhang et al., 2014; Rodrigues et al., 2017; Zarrinpar et al., 2018). Accordingly, we explored the source of Nsp15 inhibitors in the mouse intestine from the following three aspects: antibiotics themselves, intestinal microbiota, and microbiota-involved host metabolism.

To analyze the effect of antibiotics on Nsp15 activity, we conducted *in vitro* assays on an antibiotic cocktail at 1×, 10×, and 100× doses used in animal experiments. The results showed that the antibiotic cocktail had no effect on the activity of Nsp15, regardless of the dosage (**Supplementary Figure 1**). Therefore, we turned to examining the association of gut microbiota with Nsp15 inhibitors. Since antibiotic treatment is known to cause microbiome depletion (Xiao et al., 2018; Zarrinpar et al., 2018), we evaluated the perturbation of gut microbiota in mice by the antibiotics used in this study.

**Figure 3:**
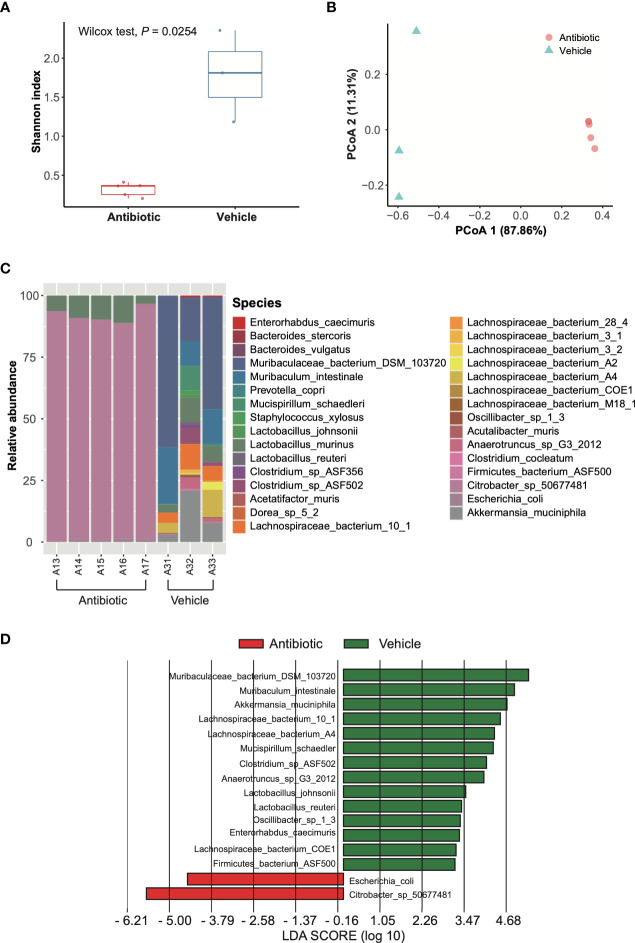
Analysis of gut microbial composition based on metagenomic data. **(A)** Shannon index. Red and blue boxes indicate antibiotic group and vehicle group, respectively. Wilcoxon test was applied to identify the difference. **(B)** PCoA based on Bray-Curtis dissimilarity index. The red circle and blue triangles indicate antibiotic individuals and vehicle individuals, respectively. The differences were compared using PERMANOVA with 9,999 permutations. **(C)** Taxonomic composition at species level. A13-A17 are antibiotic-treated individuals, while A31-A33 are vehicle individuals. **(D)** LEfSe results for species comparing the antibiotic group and vehicle group. The LDA scores > 2 are listed.

We assessed the impact of antibiotic treatment on the composition of gut microbiome by comparing the metagenome of fecal samples from antibiotic-treated mice and vehicle-treated mice. Clean reads generated from the stool-extracted DNA were subjected to quantitative profiling of the taxonomic composition of all samples. The assessment of α-diversity revealed significantly lower within-group diversity in antibiotic-treated mice compared to vehicle-treated mice (*p*=0.0254) (**Figure 3A**), while β-diversity measure detected a significant difference in taxonomic composition between antibiotic and vehicle groups (*p*=0.0193) (**Figure 3B**). The analyses of taxonomic composition at the species level suggested that the microbiome of the antibiotic-treated mice was dominated by *Citrobacter* spp. (91.19%), *Lactobacillus murinus* (7.93%), and *Escherichia coli* (0.87%), while the bacterial species that were abundant in vehicle-treated mice were *Muribaculum* spp., *Lactobacillus* spp., *Clostridium* spp., and *Lachnospiraceae* spp. (**Figure 3C**). However, only *Citrobacter* spp. and *E. coli* were enriched in antibiotic-treated mice, as revealed by analyses with LEfSe (**Figure 3D**).

**Figure 4:**
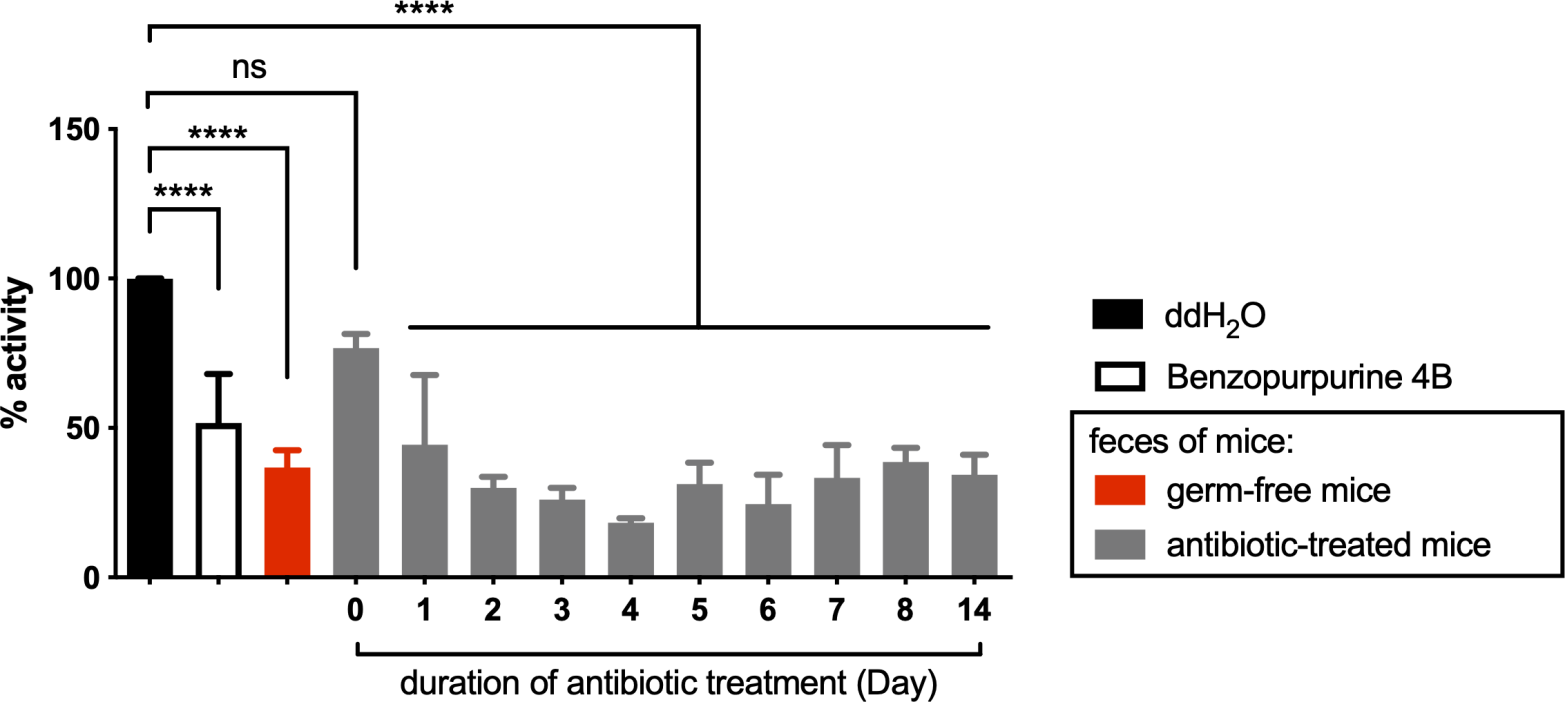
Nsp15 inhibitory activity in the intestine was associated with the impaired microbiota under antibiotic treatment. Supernatant of fecal homogenate of germ-free and antibiotic-treated mice were subjected to *in vitro* assays for the inhibitory effect on Nsp15. The reaction systems with ddH_2_O and Benzopurpurine 4B instead of the sample to be tested were negative and positive controls, respectively. Relative activity (% activity) was calculated for each sample by comparing with negative control. Values expressed are means ± S.D. from three independent experiments for no less than three biological replicates. Significance was determined by One-way ANOVA; *p*-value: ns, not significant, ****, < 0.0001.

We hypothesized that bacteria enriched by antibiotic treatment have an inhibitory effect on Nsp15 activity, and isolated these bacterial species from mouse feces to determine the impact of their bacterial cultures on Nsp15 activity *in vitro*. However, results on the most abundant species, *Citrobacter* spp., rejected the above hypothesis as its pure cultures obtained under aerobic and anaerobic conditions or co-culture with mouse feed, did not inhibit Nsp15 activity (**Supplementary Figure 2**). These results suggest that the bacteria in relatively high abundance after antibiotic treatment is not the source of Nsp15 inhibition in the mouse gut and, conversely, the Nsp15 inhibitor may be associated with the altered host metabolism due to the depleted bacteria. To verify the association between Nsp15 inhibition and microbiome depletion, we examined the inhibition of Nsp15 activity in the feces of germ-free mice. As expected, fecal supernatant from germ-free mice reduced the activity of Nsp15 by 67%, while antibiotic treatment resulted in a stable level of Nsp15 inhibition in mouse feces after two days of treatment (**Figure 4**). This result suggested that the missing microbiome play a key role in the inhibition of Nsp15 activity and that they likely act by affecting host metabolism.

### Accumulation of Primary Bile Acids Due to Loss of BSH Activity in the Gut Microbiome is a Source of Nsp15 Inhibitors

To test the above hypothesis, we further identified the pathways that were significantly reduced in the gut of antibiotic-treated mice but were enriched in that of the vehicle mice using LEfSe, with a particular focus on pathways related to microbiota-derived metabolites and microbiota-host co-metabolites. Although metagenomic data revealed global changes in host metabolism with microbiome depletion (**Supplementary Figure 3**), based on literature, we believed that these changes were associated with impaired metabolism of food-derived plant natural products and bile acids (Quinn et al., 2020). It has been reported that after antibiotic treatment, significant changes in metabolism occur in the intestine of mice: carbohydrates and sugar alcohols other than glucose and mannose is increased, fatty acid metabolism is inhibited, and the availability of short-chain fatty acids (SCFA) is decreased, a shift in amino acid metabolism is observed, the content of amino acids, vitamins, and carboxylic acids is decreased, and the ratio of primary to secondary bile acids is extremely high (Theriot et al., 2014; Rodrigues et al., 2017; Zarrinpar et al., 2018; Gutierrez et al., 2019). Our enrichment analysis agreed with these ideas, showing that the antibiotic treatment affected several metabolism-related functions of the gut microbiota, including the metabolism of bile acids, carbohydrates, fatty acids, amino acids, and vitamins (**Table S2**, **Supplementary Figure 3**).

**Figure 5:**
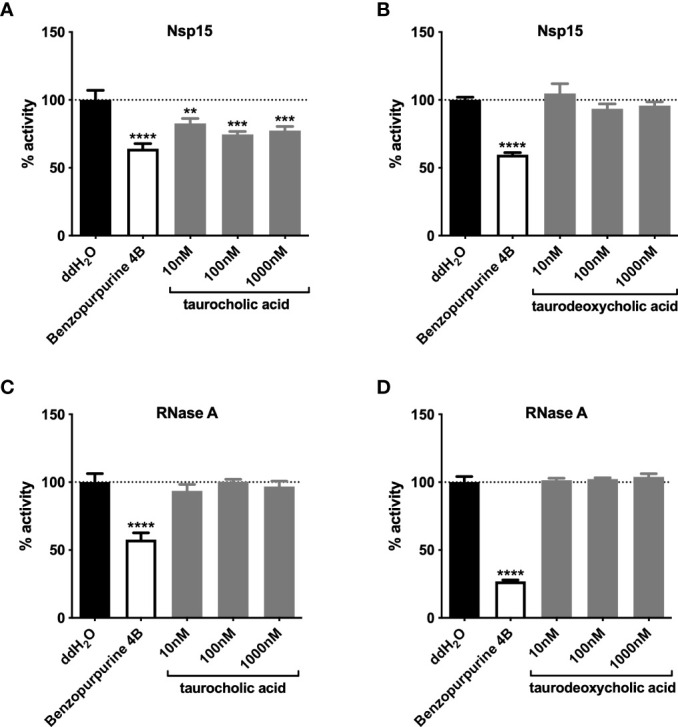
Primary bile acids inhibit Nsp15 activity. The effects of primary **(A)** and secondary **(B)** bile acids on the enzymatic activity of Nsp15 were examined. To verify the action specificity of bile acids on Nsp15, further attempts were made to test the inhibition on RNase A activity by primary **(C)** and secondary **(D)** bile acids, with each reaction system comprising 35 nM of RNase A. Reaction systems containing ddH2O or Benzopurpurine 4B rather than the sample to be tested were used as negative and positive controls, respectively. Relative activity (% activity) was calculated for each sample by comparison with the negative control. Values expressed are means ± S.D. from three independent experiments. Significance was determined by One-way ANOVA; *p*-value: **, < 0.01, ***, < 0.001, ****, < 0.0001.

Since carbohydrates and primary bile acids were metabolites enriched in the mouse gut after antibiotic treatment (Rodrigues et al., 2017; Zarrinpar et al., 2018), we examined the inhibition of Nsp15 activity *in vitro* by commercially available carbohydrates and by taurocholic acid (TCA), a primary bile acid with increased levels in antibiotic-treated mice. The results showed that carbohydrates such as fructose, cellobiose, and maltose had no impact on Nsp15 activity (**Supplementary Figure 4A**), while low doses of TCA (10 nM~1000 nM) could inhibit Nsp15 activity (**Figure 5A**). In contrast, taurodeoxycholic acid (TDCA), which is the secondary bile acid derived from TCA, had no inhibitory effect by Nsp15 (**Figure 5B**). To exclude the reduction of Nsp15 activity as a result of non-targeted aggregation of the protein by TCA (Cremers et al., 2014), we tested the inhibitory effect by TCA or TDCA on RNase A instead of Nsp15. The results showed that neither TCA (10 nM~1000 nM) nor TDCA (10 nM~1000 nM) disrupted substrate from cleavage by RNase A (**Figures 5C, D**). Further, another primary bile acid, chenodesoxycholic acid (CDCA), was examined on Nsp15 activity inhibition and we found that certain doses of CDCA (5 µM~50 µM) significantly inhibited Nsp15 activity (**Supplementary Figure 4B**). These results supported the idea that primary bile acids, inhibit Nsp15 activity.

The trend of primary bile acid accumulation in bile acid metabolism was supported by the enrichment analysis of functional genes annotated from fecal metagenomes. Based on gene family annotation against the KEGG Orthology (KO) database, K01442 (choloylglycine hydrolase, also known as bile salt hydrolases (BSHs); EC 3.5.1.24), K22605 (3alpha-hydroxycholanate dehydrogenase (NADP+)), and K15873 (7beta-hydroxy-3-oxochol-24-oyl-CoA 4-desaturase) were identified in the vehicle group, while K00076 (7-alpha-hydroxysteroid dehydrogenase) was found in both antibiotic and vehicle-treated mice (**Table S3**). K01442, contributed by *Lactobacillus johnsonii* and *Muribaculum intestinale* that were enriched in vehicle-treated group in this study, is the only KO that could function in both primary and secondary bile acid biosynthesis and was significantly lower in the antibiotic group compared to the vehicle group (*p*=0.0168) (**Supplementary Figure 5**). The other three KOs that only play a role in the secondary bile acid biosynthesis pathway did not differ significantly between the antibiotic and vehicle group. Due to the low abundance of K01442 (BSHs), the abundance of both primary bile acid biosynthesis and secondary bile acid biosynthesis pathways were significantly lower in antibiotic group (*p*= 0.0168 and 0.0358, respectively) (**Table S3**). However, considering microbial K01442 (BSHs) present in the gut can catalyze the hydrolysis of conjugated bile salts into deconjugated bile acids (Coleman and Hudson, 1995), our results suggested that antibiotic treatment would induce a shift in the gut microbiota composition and impair the BSH activity of microbiota thus inhibiting the secondary bile acid biosynthesis and accumulating primary bile acids, leading to an inhibitory effect on Nsp15 in the mouse GI tract.

## Discussion

The COVID-19 pandemic caused by SARS-CoV-2 is rapidly spreading and evolving around the world. Though conventional antiviral drugs (Holshue et al., 2020; McKee et al., 2020; NIH, 2022), traditional Chinese medicine (Yang et al., 2020; Wang and Yang, 2021; Shi et al., 2022), antibiotics (Mirzaei et al., 2020; Popp et al., 2021), and vaccines (Poland et al., 2020; Eyre et al., 2022; WHO, 2022a) have been applied in COVID-19 clinical therapy, the screening for drugs targeting SARS-CoV-2 critical proteins remains a long and arduous process

### Primary Bile Acids Inhibit Nsp15 Activity

A considerable portion of COVID-19 patients showed liver damage, which might result from direct cholangiocyte injury and consequent bile acid accumulation induced by viral infection (Zhao B. et al., 2020). Bile acids are important substances involved in the regulation of the hepatic-intestinal axis. The liver converts cholesterol into primary bile acids, which are subsequently transported into bile and stored in the gallbladder until released into the intestine. The primary bile acids can be further metabolized by the gut microbiota in the jejunum or terminal ileum or can be circulated through the portal vein reabsorption to the liver (Ridlon et al., 2016).

The human intestinal microbiome can be disrupted by antibiotics (Adamsson et al., 1999; Jernberg et al., 2007; Jakobsson et al., 2010; Subramanian et al., 2014; de Gunzburg et al., 2018) that lead to the imbalance of microbiome-bile acid pool (Theriot et al., 2014; Kuno et al., 2018; Behr et al., 2019). After antibiotic treatment, the fecal microbial communities were dominated by Firmicutes and Proteobacteria (Jakobsson et al., 2010), which is consistent with our result that the microbiota of the antibiotic-treated mice was dominated by *Citrobacter* spp., *Lactobacillus murinus*, and *Escherichia coli* (**Figure 3C**). Antibiotic-induced microbiome depletion alters the production of bile acids (Ridlon et al., 2014). In particular, cholic acid (CA) and β-muricholic acid (bMCA) are significantly reduced, with an almost two-fold increase in TCA, a three-fold increase in tauro-β-muricholic acid (TbMCA), and a three-fold increase in taurochenodeoxycholic acid (TCDCA) (Zarrinpar et al., 2018). In this study, we found that antibiotic-induced alteration of gut microbiota led to an inhibitory effect on the activity of SARS-CoV-2 Nsp15 *via* accumulating primary bile acids, suggesting that Nsp15 is a potential drug target for therapies of coronavirus. However, the potential of TCA in the therapy of COVID-19 infection should be investigated in further studies. Follow-up studies could initially focus on assessing the antiviral effects of TCA using cellular models (Canal et al., 2021; Kim et al., 2021).

### The Use of Dual-Mechanism Antibiotics in the COVID-19 Pandemic

It is well known that a subset of patients with severe SARS-CoV-2 infection commonly develop a clinically severe hyperinflammatory state or cytokine storm, with respiratory distress syndrome (Ruan et al., 2020; Zhou et al., 2020). Over 70% of the patients with COVID-19 received antibiotics due to symptoms similar to bacterial pneumonia or secondary bacterial infections (Knight et al., 2021; Langford et al., 2021). However, numerous studies suggest that antibiotic prescriptions are excessively applied in the treatment on COVID-19 infection (Lai et al., 2020; Langford et al., 2020; Youngs et al., 2020; RECOVERY Collaborative Group, 2021; PRINCIPLE Trial Collaborative Group, 2021). The chronic overuse of antibiotics not only disrupts the composition of microbiota (Subramanian et al., 2014; Theriot et al., 2016; Kang et al., 2022), but also expands the spectrum of bacterial resistance to antibiotics, posing a threat to human health (Llor and Bjerrum, 2014; Huang et al., 2020). Moreover, antibiotic treatment leads to the imbalance of the microbiome-bile acid pool. In the present study, we found that the composition of gut microbiota changed under antibiotic treatment, as evidenced by a relative increase in *Citrobacter* and *Lactobacillus* bacteria, an absence of anti-inflammatory bacteria *Akkermansia muciniphila*, and the bile acid metabolism-related bacterias *Lactobacillus* and *Clostridium*. Although antibiotic treatment is likely to affect the anti-inflammation and bile acid metabolism in the gut, the resulting accumulation of primary bile acids contributes to the inhibitory effect on SARS-Cov-2 Nsp15, according to this study. The above results suggest that antibiotics may not only make the gut microbiome alterative, but also alter the bile acid metabolism of patients, and thus, protect the patients from SARS-CoV-2 infection.

### The Potential of Bile Acids for the Development of COVID-19 Inhibitors

Based on its cytoprotective, anti-inflammatory, and immunomodulatory properties, in particular, its beneficial effects of ursodeoxycholic acid (UDCA) in respiratory diseases such as effective modulation of Th2-derived cytokines and inhibition of apoptosis of airway epithelial cells in mouse models of chronic asthma, along with the stimulation of alveolar fluid clearance in a rat model of acute respiratory distress syndrome *via* the ALX/cAMP/PI3K pathway, the FDA-approved drug UDCA has been suggested for COVID-19 treatment (Abdulrab et al., 2020; Hirayama et al., 2021). Moreover, bile acids play an important regulatory role in viral replication (Schupp and Graf, 2014). For example, the replication of influenza A virus and hepatitis B virus (HBV) is inhibited by bile acids (Hassan et al., 2012; Mouzannar et al., 2019). Furthermore, bile acids have been found to enhance genotype 1 hepatitis C virus replication through FXR (Scholtes et al., 2008). In a recent study, primary and secondary bile acids and their amino derivatives, such as glycoursodeoxycholic acid, and semisynthetic derivatives, such as obeticholic acid, are found to reduce the binding between the RBD region of SARS-CoV-2 S protein and human ACE2 protein (Carino et al., 2020). Gut microbiota have been suggested as possible indicators of SARS-CoV-2 infection and to be involved in anti-infection (Yeoh et al., 2021; Zuo et al., 2021b). Therefore, it is important and valuable to explore the intestinal resources for applications in COVID-19 therapy. This study revealed that antibiotic treatment can induce a shift in the gut microbiota and lead to impaired microbial BSH activity. This alteration in microbiota-host co-metabolites was characterized by the blockage of secondary bile acid biosynthesis and the accumulation of primary bile acids, which was identified as Nsp15 inhibitors in this study. These findings will benefit the development of choline and its derivatives as SARS-CoV-2 inhibitors.

## Materials and Methods

### Strains and Plasmids

Bacterial strains and plasmids used in this study are listed in **Table S1**. All *Escherichia coli* strains used in this study were propagated in LB media containing appropriate antibiotics (100 μg/ml for ampicillin) at 37°C unless otherwise noted. The Endoribonuclease (*nsp15*) gene was optimized with *E. coli* codons and cloned into pET32a (+) plasmid. The pET32a (+) plasmid was transformed into *E. coli* BL21(DE3) cells to obtain a high yield of recombinant Nsp15 protein.

### Expression of Nsp15

Recombinant SARS-CoV-2 Nsp15 protein was expressed, as previously described, with slight modification (Bhardwaj et al., 2004; Fan et al., 2004). Briefly, the overnight culture of *E. coli* BL21(DE3) cells containing the pET32a (+)-*nsp15*-His plasmids was sub-cultured 1:100 in 250 ml LB containing 100 μg/ml ampicillin. Cells were grown to the optical density at 600nm of 0.9-1.0 and then induced with 1 mM Isopropyl-β-D-1 thiogalactopyranoside (IPTG). After additional incubation at 18°C for 16 h, cells were harvested by centrifugation at 4°C, 8,000*×g* for 2 min, resuspended in 10 ml of cold lysis buffer, digested with 1 mg/ml lysozyme for 30 min, and finally lysed with sonication. Cell debris was removed by centrifugation at 4°C, 10,000*×g* for 30 min. The supernatant containing Nsp15 protein (Nsp15 crude enzyme) was added with 50% glycerol and 50 mM Tris-HCl (pH 8.0), and stored at -80°C. The Nsp15 crude enzyme was purified using Ni-NTA Agarose (QIAGEN, Germany), and dialyzed overnight against 20 mM PBS (pH 7.0) buffer containing 25% glycerol by 15 KDa dialysis membrane. Protein purity was determined by 12% polyacrylamide gel electrophoresis.

### Endoribonuclease Assay

Fluorogenic RNA substrates 5’6-FAM-GAAGCGAAACCC/rU/AAG-3’BHQ1 (substrate N1), 5’6-FAM- GCGGAGCACG A/rU/CGAG - 3’BHQ1 (substrate N2), 5’6-FAM-UACGAUCG-3’BHQ1 (substrate N3) were synthesized by Tsingke Biotechnology (Beijing, China), 5’6-FAM - A/rU/AA - 3’BHQ1 (substrate N4) was prepared by Sangon Biotech (Shanghai, China). The RNA cleavage assay was performed at 30°C in the reaction buffer (50 mM Tris-HCl pH 7.5, 100 mM KCl, 5 mM MnCl_2_, 1 mM DTT) containing 1 µM fluorescent RNA substrate and 317 nM of Nsp15. Enhanced fluorescence due to cleavage of the fluorogenic substrate RNA was monitored at 518 nm with excitation at 492 nm (Bhardwaj et al., 2004), using a fluorescence plate reader (Cytation5, Bio Tek, US). Doubling of fluorescence intensity per minute in a reaction system containing the fluorescent RNA substrate N4 and SARS-CoV-2 Nsp15 compared to a reaction system containing only substrate was defined as one activity unit (U). The reaction system containing Benzopurpurine 4B (final concentration of 1 mM) was used as positive control in examination as previously described (Ortiz-Alcantara, 2010).

### Animal Assay

The antibiotic-treated adult mouse model was used to assess the impact of the intestinal environment on SARS-CoV-2 protein activity. We used male mice in this study in order to minimize potential experimental error. Briefly, six-week-old C57BL/6J males were randomly assigned to each experimental group (6 mice/group) and then supplied with drinking water with or without antibiotic cocktail (0.1 g/L vancomycin, 0.2 g/L neomycin, 0.2 g/L ampicillin, 0.2 g/L metronidazole) and 0.2 g/L aspartame for two weeks. To investigate the interaction between gut microbiota and CPMs used in the clinical treatment of COVID-19, the drug was administered or not by gavage once daily for the last 7 days using either LHQWC and JHQGG. Fecal pellets were collected from each mouse daily throughout the experiment. Samples for metagenomic shotgun sequencing were collected at day 14 and directly frozen at -80°C, while samples for endoribonuclease assay and bacteria isolation were collected at the indicated time-point and homogenized with ddH_2_O. Germ-free C57BL/6J mice fecal samples were obtained from the College of Animal Sciences and Technology at Huazhong Agricultural University in Wuhan, China.

### Metagenomic Shotgun Sequencing and Analysis

We used the phenol/chloroform/isoamyl alcohol method to extract microbial DNA from fecal pellets of antibiotic-treated and vehicle-treated mice (Zoetendal et al., 2006). DNA degradation and potential contamination was monitored on 1% agarose gels and the DNA concentration was measured using Qubit^®^ dsDNA BR Assay Kit in Qubit^®^ 2.0 Fluorometer (Life Technologies, CA, USA). The DNA that passed quality control was then subjected to library construction using the TruSeq DNA HT Sample Prep Kit. Paired-end 150 metagenomic shotgun sequencing (mean of 39,350,387 reads per sample) was performed on the Illumina Hi-Seq platform. The sequencing reads subjected to quality control using Kneaddata (https://github.com/biobakery/biobakery/wiki/kneaddata) and host sequences were removed based on the mouse_C57BL_6NJ database. High quality sequences were used for quantitative profiling of the taxonomic composition of the microbial communities of all samples using MetaPhlAn 3.0 (Segata et al., 2012) (https://github.com/biobakery/MetaPhlAn/wiki/MetaPhlAn-3.0), whereas HUMAnN 3.0 (Franzosa et al., 2018) (https://huttenhower.sph.harvard.edu/humann) was used to profile gene-family abundances. Gene families determined by UniRef were mapped to the KEGG Orthology (KO) database and were grouped into the KEGG pathway. The alpha diversity index (Shannon index) and Bray-Curtis dissimilarities were calculated using Vegan package in R. Statistical analysis was performed on Shannon index and KO between groups using a non-parametric Wilcoxon test with a *p*-value < 0.05. Principal coordinate analysis (PCoA) was performed on beta diversity based on Bray-Curtis distances and the differences were compared using permutational multivariate ANOVA (PERMANOVA) with 9,999 permutations. LEfSe (Segata et al., 2011) was used to quantitate differential taxonomic abundance and pathway abundance using a Kruskal-Wallis test with α value of 0.05 and a log linear discriminant analysis (LDA) score cut-off of 2.

## Data Availability Statement

The metagenomic data have been deposited at Sequence Read Archive database of NCBI with the accession number PRJNA734104.

## Ethics Statement

The animal study was reviewed and approved by The Ethical Committee of Huazhong University of Science and Technology. (Permit Number: SYXK (E) 2016-0057).

## Author Contributions

ZL, YM, and XF conceived the study and designed the experiment. ML, YD, XY, XW, and GC performed the experiment. YSD, LH, YC, PW, HW, and MN analyzed the data. YM, ML, YSD, XY, GC, and ZQ wrote the manuscript. All authors critically revised the manuscript. All authors read and approved the final manuscript.

## Funding

This work was supported by the National Key R&D Program of China (2019YFA0905600), and the HUST COVID-19 Rapid Response Call (2020kfyXGYJ047), the Specific Fund of State Key Laboratory of Dampness Syndrome of Chinese Medicine (SZ2021ZZ28), and the Science and Technology Planning Project of Guangdong Province (2020B1111100005).

## Conflict of Interest

YSD and LH are employed by KMHD, Shenzhen, China.

The remaining authors declare that the research was conducted in the absence of any commercial or financial relationships that could be construed as a potential conflict of interest.

## Publisher’s Note

All claims expressed in this article are solely those of the authors and do not necessarily represent those of their affiliated organizations, or those of the publisher, the editors and the reviewers. Any product that may be evaluated in this article, or claim that may be made by its manufacturer, is not guaranteed or endorsed by the publisher.

## Supplementary Material

Supplementary Figure 1 Antibiotics had no impact on Nsp15 activity. The effect of antibiotics on Nsp15 activity was evaluated *in vitro* at 1-100 times the dose used in animal experiments (gray bars). The reaction systems with ddH_2_O (black bar) and Benzopurpurine 4B (white bar), instead of the sample to be tested, were negative and positive controls, respectively. Relative activity (% activity) was calculated for each sample by comparing with the negative control. Values expressed are means ± S.D. from three independent experiments. Significance was determined by One-way ANOVA; *p*-value: ****, < 0.0001.

Supplementary Figure 2 Antibiotic-induced enrichment of *Citrobacter* spp. did not relate to the Nsp15 inhibition in feces. Supernatant of *Citrobacter* spp. cultures was prepared aerobically (gray bars) or anaerobically (white bars filled with grey slashes) in LB with or without feed, respectively, to assay the effect on Nsp15 activity *in vitro*. The reaction systems with ddH_2_O and Benzopurpurine 4B, instead of the sample to be tested, were used as negative and positive controls, respectively. Relative activity (% activity) was calculated for each sample by comparing with the negative control. Values expressed are means ± S.D. from three experiments. Significance of difference was determined by One-way ANOVA; *p*-value: ****, < 0.0001.

Supplementary Figure 3 Heatmap of pathways related to metabolism enriched in the vehicle group. Pathways enriched in the vehicle group were identified using LEfSe with LDA score >2 and Kruskal-Wallis test *p*-value < 0.05. The color of cells from blue to red corresponds to the scaling for the relative abundance of pathways from low to high. Pathways that were regrouped to carbohydrate and glycan metabolism, vitamin metabolism, bile acid metabolism, amino acid metabolism, fatty acids, and other lipid metabolism, etc. were labeled with different colors. Both rows and columns are clustered using correlation distance and complete linkage.

Supplementary Figure 4 The effect of antibiotic-enriched metabolites on Nsp15 enzyme activity. **(A)** Antibiotic-enriched oligosaccharide metabolites did not inhibit Nsp15 enzyme activity. The highly abundant oligosaccharide metabolites such as fructose, cellobiose, and maltose were subjected to *in vitro* assay for the inhibitory effect on Nsp15. The reaction systems with ddH_2_O and Benzopurpurine 4B, instead of the sample to be tested, were negative and positive controls, respectively. Relative activity (% activity) was calculated for each sample by comparing with negative control. Values expressed are means ± S.D. from three experiments. Significance was determined by One-way ANOVA; *p*-value: ****, < 0.0001. **(B)** The effect of primary bile acids CDCA at different concentrations effect of Nsp15 activity. The reaction systems with ddH_2_O and Benzopurpurine 4B, instead of the sample to be tested, were negative and positive controls, respectively. Relative activity (% activity) was calculated for each sample by comparing with negative control. Values expressed are means ± S.D. from three independent experiments. Significance was determined by One-way ANOVA; *p*-value: ****, < 0.0001.

Supplementary Figure 5 Enrichment analysis of functional genes annotated from metagenomes of mouse fecal samples. Gene families were mapped to the KO database and screened out KOs in the bile acid metabolism pathway (primary bile acid biosynthesis, KEGG map00120; secondary bile acid biosynthesis, KEGG map00121). K01442 (choloylglycine hydrolase; EC 3.5.1.24), K22605 (3alpha-hydroxycholanate dehydrogenase (NADP+)), K00076 (7-alpha-hydroxysteroid dehydrogenase), and K15873 (7beta-hydroxy-3-oxochol-24-oyl-CoA 4-desaturase) were annotated as a result. Red and blue boxes indicated the antibiotic group and vehicle group, respectively. The Wilcoxon test was applied to identify the differentially features. *p*-value: *, < 0.05.

Table S2 Annotation of gene families related to metabolism.

Table S3 Annotation of gene families related to bile acid metabolism.
