# Supplementary figures and images for: Antibiotic-Induced Primary Biles Inhibit SARS-CoV-2 Endoribonuclease Nsp15 Activity in Mouse Gut
Source: Front Cell Infect Microbiol. 2022 Jul 28;12:896504. doi: 10.3389/fcimb.2022.896504 (PMC9366059; doi:10.3389/fcimb.2022.896504)

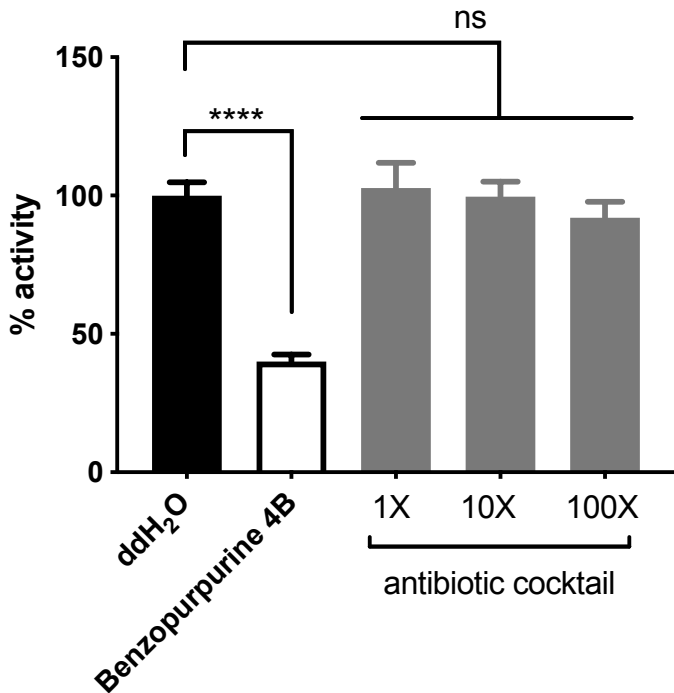

Supplement: Supplementary Figure 1 — Antibiotics had no impact on Nsp15 activity. The effect of antibiotics on Nsp15 activity was evaluated in vitro at 1-100 times the dose used in animal experiments (gray bars). The reaction systems with ddH2O (black bar) and Benzopurpurine 4B (white bar), instead of the sample to be tested, were negative and positive controls, respectively. Relative activity (% activity) was calculated for each sample by comparing with the negative control. Values expressed are means ± S.D. from three independent experiments. Significance was determined by One-way ANOVA; p-value: ****, < 0.0001. [file DataSheet_1.pdf]

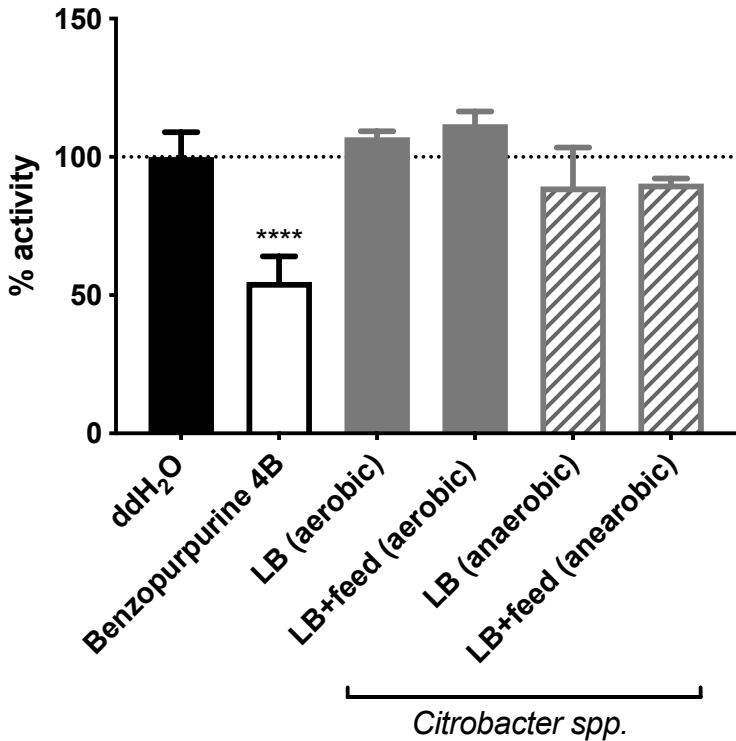

Supplement: Supplementary Figure 2 — Antibiotic-induced enrichment of Citrobacter spp. did not relate to the Nsp15 inhibition in feces. Supernatant of Citrobacter spp. cultures was prepared aerobically (gray bars) or anaerobically (white bars filled with grey slashes) in LB with or without feed, respectively, to assay the effect on Nsp15 activity in vitro. The reaction systems with ddH2O and Benzopurpurine 4B, instead of the sample to be tested, were used as negative and positive controls, respectively. Relative activity (% activity) was calculated for each sample by comparing with the negative control. Values expressed are means ± S.D. from three experiments. Significance of difference was determined by One-way ANOVA; p-value: ****, < 0.0001. [file DataSheet_2.pdf]

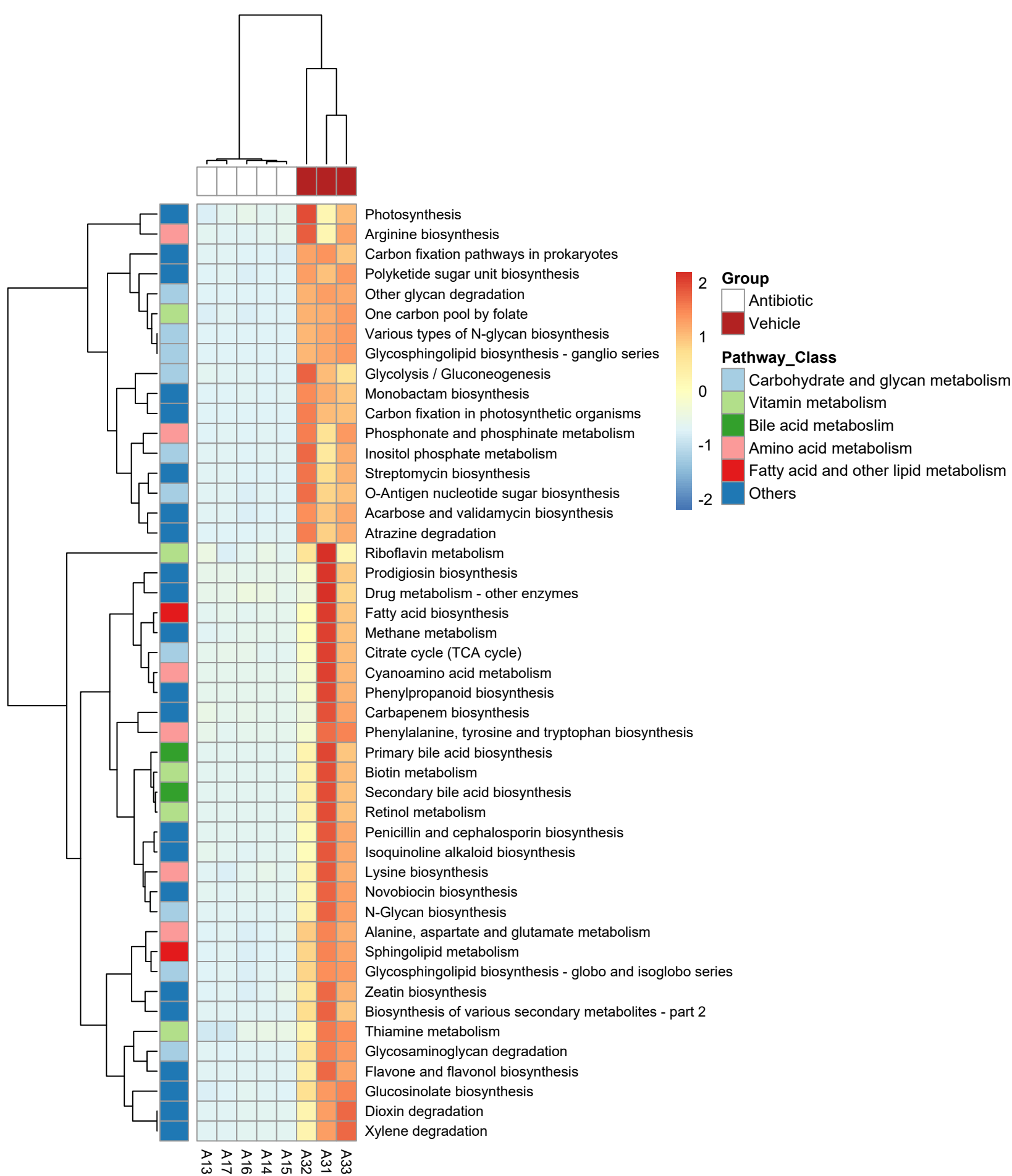

Supplement: Supplementary Figure 3 — Heatmap of pathways related to metabolism enriched in the vehicle group. Pathways enriched in the vehicle group were identified using LEfSe with LDA score >2 and Kruskal-Wallis test p-value < 0.05. The color of cells from blue to red corresponds to the scaling for the relative abundance of pathways from low to high. Pathways that were regrouped to carbohydrate and glycan metabolism, vitamin metabolism, bile acid metabolism, amino acid metabolism, fatty acids, and other lipid metabolism, etc. were labeled with different colors. Both rows and columns are clustered using correlation distance and complete linkage. [file DataSheet_3.pdf]

**(A)** **Oligosaccharides**

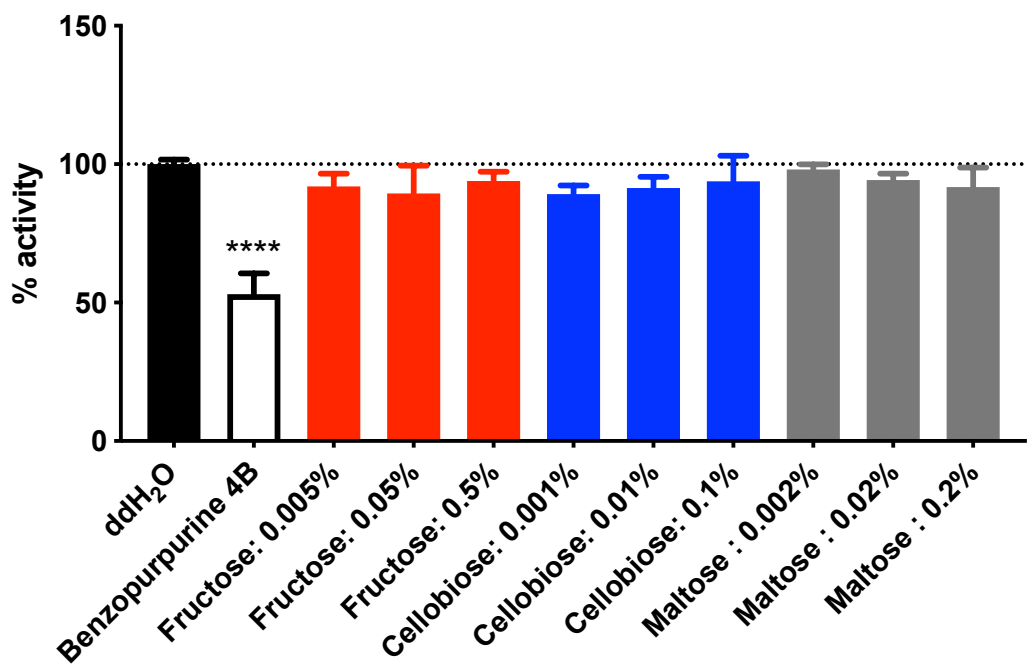

**(B)** **CDCA**

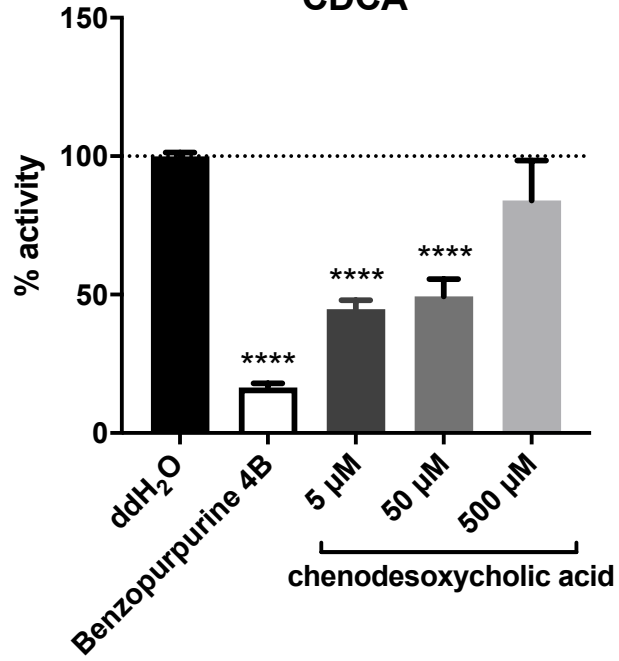

Supplement: Supplementary Figure 4 — The effect of antibiotic-enriched metabolites on Nsp15 enzyme activity. (A) Antibiotic-enriched oligosaccharide metabolites did not inhibit Nsp15 enzyme activity. The highly abundant oligosaccharide metabolites such as fructose, cellobiose, and maltose were subjected to in vitro assay for the inhibitory effect on Nsp15. The reaction systems with ddH2O and Benzopurpurine 4B, instead of the sample to be tested, were negative and positive controls, respectively. Relative activity (% activity) was calculated for each sample by comparing with negative control. Values expressed are means ± S.D. from three experiments. Significance was determined by One-way ANOVA; p-value: ****, < 0.0001. (B) The effect of primary bile acids CDCA at different concentrations effect of Nsp15 activity. The reaction systems with ddH2O and Benzopurpurine 4B, instead of the sample to be tested, were negative and positive controls, respectively. Relative activity (% activity) was calculated for each sample by comparing with negative control. Values expressed are means ± S.D. from three independent experiments. Significance was determined by One-way ANOVA; p-value: ****, < 0.0001. [file DataSheet_4.pdf]

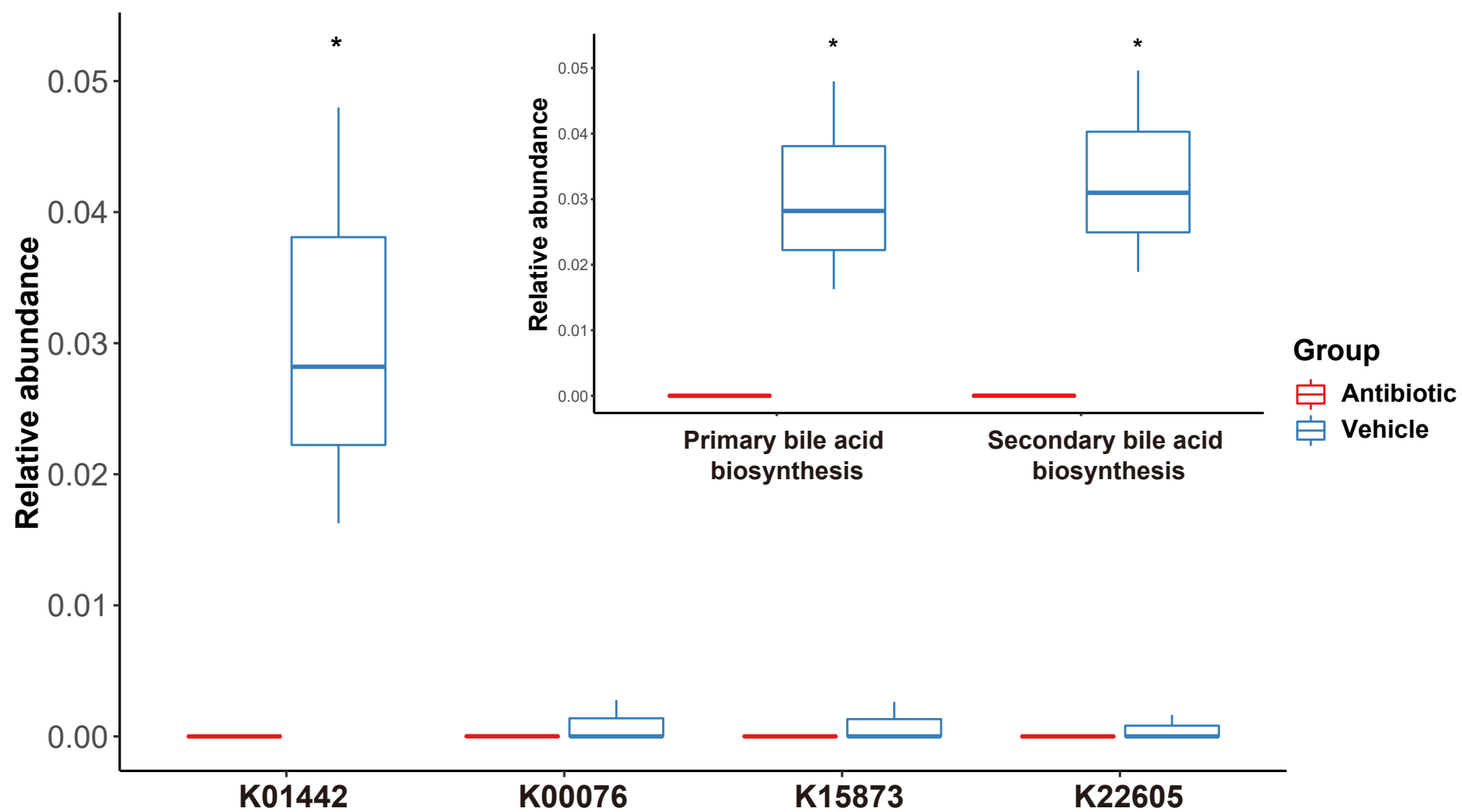

Supplement: Supplementary Figure 5 — Enrichment analysis of functional genes annotated from metagenomes of mouse fecal samples. Gene families were mapped to the KO database and screened out KOs in the bile acid metabolism pathway (primary bile acid biosynthesis, KEGG map00120; secondary bile acid biosynthesis, KEGG map00121). K01442 (choloylglycine hydrolase; EC 3.5.1.24), K22605 (3alpha-hydroxycholanate dehydrogenase (NADP+)), K00076 (7-alpha-hydroxysteroid dehydrogenase), and K15873 (7beta-hydroxy-3-oxochol-24-oyl-CoA 4-desaturase) were annotated as a result. Red and blue boxes indicated the antibiotic group and vehicle group, respectively. The Wilcoxon test was applied to identify the differentially features. p-value: *, < 0.05. [file DataSheet_5.pdf]
