# Supplementary material for: Antibiotic-Induced Primary Biles Inhibit SARS-CoV-2 Endoribonuclease Nsp15 Activity in Mouse Gut
Source: Front Cell Infect Microbiol. 2022 Jul 28;12:896504. doi: 10.3389/fcimb.2022.896504 (PMC9366059; doi:10.3389/fcimb.2022.896504)
Supplement: Supplementary file 6 [file DataSheet_6.pdf]

Table S1. Strains and plasmids used in this study.

Strain or plasmid	Genotype or description	Source
**Strains**		
E. coli BL21(DE3)	F^- *ompT hsdS*$_B$ (r_B^- m_B^-) *gal dcm* (DE3)	Lab stock
**Plasmids**		
pET-32a(+)-NSP15	pET-32a(+) containing the SARS-CoV-2 endoribonuclease (Nsp15) gene with *E. coli* codon optimization, Amp^r	Dr. Shengce Tao
