# Supplementary material for: Antibiotic-Induced Primary Biles Inhibit SARS-CoV-2 Endoribonuclease Nsp15 Activity in Mouse Gut
Source: Front Cell Infect Microbiol. 2022 Jul 28;12:896504. doi: 10.3389/fcimb.2022.896504 (PMC9366059; doi:10.3389/fcimb.2022.896504)
Supplement: Table S2 — Annotation of gene families related to metabolism. [file DataSheet_7.pdf]

Table S2. Annotation of gene families related to metabolism.

Bile acid metaboslim

ID	Enriched_group	LDA_score	P_value	PathwayName	Class	Group
**ko00120**	Vehicle	2.11330766	0.010414995	Primary bile acid biosynthesis	Lipid metabolism	Metabolism
**ko00121**	Vehicle	2.14323236	0.024480834	Secondary bile acid biosynthesis	Lipid metabolism	Metabolism
**ko00430**	Antibiotic	2.700508977	0.025347319	Taurine and hypotaurine metabolism	Metabolism of other amino acids	Metabolism

Fatty acid and other lipid metabolism

ID	Enriched_group	LDA_score	P_value	PathwayName	Class	Group
**ko00061**	Vehicle	3.611267784	0.025347319	Fatty acid biosynthesis	Lipid metabolism	Metabolism
**ko00600**	Vehicle	2.990671667	0.025347319	Sphingolipid metabolism	Lipid metabolism	Metabolism
**ko00071**	Antibiotic	3.251740122	0.025347319	Fatty acid degradation	Lipid metabolism	Metabolism
**ko00072**	Antibiotic	2.691090141	0.025347319	Synthesis and degradation of ketone bodies	Lipid metabolism	Metabolism
**ko00561**	Antibiotic	2.805514369	0.025347319	Glycerolipid metabolism	Lipid metabolism	Metabolism
**ko00564**	Antibiotic	3.469324214	0.025347319	Glycerophospholipid metabolism	Lipid metabolism	Metabolism
**ko00565**	Antibiotic	2.28865822	0.025347319	Ether lipid metabolism	Lipid metabolism	Metabolism
**ko00590**	Antibiotic	2.637396724	0.025347319	Arachidonic acid metabolism	Lipid metabolism	Metabolism
**ko00591**	Antibiotic	2.306592304	0.025347319	Linoleic acid metabolism	Lipid metabolism	Metabolism
**ko00592**	Antibiotic	2.898489066	0.025347319	alpha-Linolenic acid metabolism	Lipid metabolism	Metabolism
**ko01040**	Antibiotic	2.953178681	0.021946771	Biosynthesis of unsaturated fatty acids	Lipid metabolism	Metabolism

Amino acid metabolism

ID	Enriched_group	LDA_score	P_value	PathwayName	Class	Group
**ko00220**	Vehicle	3.048898876	0.025347319	Arginine biosynthesis	Amino acid metabolism	Metabolism
**ko00250**	Vehicle	3.062592371	0.025347319	Alanine, aspartate and glutamate metabolism	Amino acid metabolism	Metabolism
**ko00300**	Vehicle	2.939196879	0.025347319	Lysine biosynthesis	Amino acid metabolism	Metabolism
**ko00400**	Vehicle	2.995901329	0.025347319	Phenylalanine, tyrosine and tryptophan biosynthesis	Amino acid metabolism	Metabolism
**ko00440**	Vehicle	2.454669868	0.025347319	Phosphonate and phosphinate metabolism	Metabolism of other amino acids	Metabolism
**ko00460**	Vehicle	3.383515374	0.025347319	Cyanoamino acid metabolism	Metabolism of other amino acids	Metabolism
**ko00260**	Antibiotic	3.264396971	0.025347319	Glycine, serine and threonine metabolism	Amino acid metabolism	Metabolism
**ko00270**	Antibiotic	2.650120718	0.025347319	Cysteine and methionine metabolism	Amino acid metabolism	Metabolism
**ko00290**	Antibiotic	2.68016333	0.025347319	Valine, leucine and isoleucine biosynthesis	Amino acid metabolism	Metabolism
**ko00310**	Antibiotic	3.415569891	0.025347319	Lysine degradation	Amino acid metabolism	Metabolism
**ko00330**	Antibiotic	2.905889209	0.025347319	Arginine and proline metabolism	Amino acid metabolism	Metabolism
**ko00350**	Antibiotic	2.676590824	0.025347319	Tyrosine metabolism	Amino acid metabolism	Metabolism
**ko00380**	Antibiotic	3.150334697	0.025347319	Tryptophan metabolism	Amino acid metabolism	Metabolism
**ko00471**	Antibiotic	2.419830076	0.025347319	D-Glutamine and D-glutamate metabolism	Metabolism of other amino acids	Metabolism
**ko00410**	Antibiotic	2.469227446	0.025347319	beta-Alanine metabolism	Metabolism of other amino acids	Metabolism
**ko00450**	Antibiotic	2.726557968	0.025347319	Selenocompound metabolism	Metabolism of other amino acids	Metabolism
**ko00473**	Antibiotic	2.401073523	0.025347319	D-Alanine metabolism	Metabolism of other amino acids	Metabolism
**ko00480**	Antibiotic	3.468977422	0.025347319	Glutathione metabolism	Metabolism of other amino acids	Metabolism

Table S2. Annotation of gene families related to metabolism. (Continued)

Carbohydrate and glycan metabolism

ID	Enriched_group	LDA_score	P_value	PathwayName	Class	Group
ko00510	Vehicle	2.231625069	0.010414995	N-Glycan biosynthesis	Glycan biosynthesis and metabolism	Metabolism
ko00511	Vehicle	3.199463618	0.024480834	Other glycan degradation	Glycan biosynthesis and metabolism	Metabolism
ko00513	Vehicle	3.000503838	0.010414995	Various types of N-glycan biosynthesis	Glycan biosynthesis and metabolism	Metabolism
ko00531	Vehicle	2.804019032	0.025347319	Glycosaminoglycan degradation	Glycan biosynthesis and metabolism	Metabolism
ko00541	Vehicle	2.957833342	0.025347319	O-Antigen nucleotide sugar biosynthesis	Glycan biosynthesis and metabolism	Metabolism
ko00603	Vehicle	2.663989851	0.025347319	Glycosphingolipid biosynthesis - globo and isoglobo series	Glycan biosynthesis and metabolism	Metabolism
ko00604	Vehicle	2.679445681	0.010414995	Glycosphingolipid biosynthesis - ganglio series	Glycan biosynthesis and metabolism	Metabolism
ko00010	Vehicle	3.531921359	0.025347319	Glycolysis / Gluconeogenesis	Carbohydrate metabolism	Metabolism
ko00020	Vehicle	2.861057782	0.025347319	Citrate cycle (TCA cycle)	Carbohydrate metabolism	Metabolism
ko00562	Vehicle	3.055359966	0.025347319	Inositol phosphate metabolism	Carbohydrate metabolism	Metabolism
ko00540	Antibiotic	3.43850187	0.025347319	Lipopolysaccharide biosynthesis	Glycan biosynthesis and metabolism	Metabolism
ko00572	Antibiotic	2.796715761	0.021946771	Arabinogalactan biosynthesis - Mycobacterium	Glycan biosynthesis and metabolism	Metabolism
ko00040	Antibiotic	3.326906113	0.025347319	Pentose and glucuronate interconversions	Carbohydrate metabolism	Metabolism
ko00053	Antibiotic	3.450585927	0.025347319	Ascorbate and aldarate metabolism	Carbohydrate metabolism	Metabolism
ko00620	Antibiotic	3.53377202	0.025347319	Pyruvate metabolism	Carbohydrate metabolism	Metabolism
ko00630	Antibiotic	3.357142784	0.025347319	Glyoxylate and dicarboxylate metabolism	Carbohydrate metabolism	Metabolism
ko00640	Antibiotic	3.587129331	0.025347319	Propanoate metabolism	Carbohydrate metabolism	Metabolism
ko00650	Antibiotic	3.27111884	0.025347319	Butanoate metabolism	Carbohydrate metabolism	Metabolism
ko00660	Antibiotic	3.165854031	0.025347319	C5-Branched dibasic acid metabolism	Carbohydrate metabolism	Metabolism

Vitamin metabolism

ID	Enriched_group	LDA_score	P_value	PathwayName	Class	Group
ko00670	Vehicle	2.92465632	0.025347319	One carbon pool by folate	Metabolism of cofactors and vitamins	Metabolism
ko00730	Vehicle	2.612533565	0.025347319	Thiamine metabolism	Metabolism of cofactors and vitamins	Metabolism
ko00740	Vehicle	2.345025228	0.025347319	Riboflavin metabolism	Metabolism of cofactors and vitamins	Metabolism
ko00780	Vehicle	3.737997894	0.025347319	Biotin metabolism	Metabolism of cofactors and vitamins	Metabolism
ko00830	Vehicle	3.06081788	0.024480834	Retinol metabolism	Metabolism of cofactors and vitamins	Metabolism
ko00130	Antibiotic	3.479994978	0.025347319	Ubiquinone and other terpenoid-quinone biosynthesis	Metabolism of cofactors and vitamins	Metabolism
ko00750	Antibiotic	2.593893676	0.025347319	Vitamin B6 metabolism	Metabolism of cofactors and vitamins	Metabolism
ko00760	Antibiotic	3.284300725	0.025347319	Nicotinate and nicotinamide metabolism	Metabolism of cofactors and vitamins	Metabolism
ko00785	Antibiotic	2.408758768	0.025347319	Lipoic acid metabolism	Metabolism of cofactors and vitamins	Metabolism
ko00860	Antibiotic	3.65021879	0.025347319	Porphyrin and chlorophyll metabolism	Metabolism of cofactors and vitamins	Metabolism

Others

ID	Enriched_group	LDA_score	P_value	PathwayName	Class	Group
ko00261	Vehicle	2.480615171	0.025347319	Monobactam biosynthesis	Biosynthesis of other secondary metabolites	Metabolism
ko00311	Vehicle	2.46685975	0.010414995	Penicillin and cephalosporin biosynthesis	Biosynthesis of other secondary metabolites	Metabolism
ko00332	Vehicle	2.277201546	0.025347319	Carbapenem biosynthesis	Biosynthesis of other secondary metabolites	Metabolism

Table S2. Annotation of gene families related to metabolism. (Continued)

Others

ID	Enriched_group	LDA_score	P_value	PathwayName	Class	Group
**ko00333**	Vehicle	2.957257625	0.025347319	Prodigiosin biosynthesis	Biosynthesis of other secondary metabolites	Metabolism
**ko00401**	Vehicle	2.47995506	0.025347319	Novobiocin biosynthesis	Biosynthesis of other secondary metabolites	Metabolism
**ko00521**	Vehicle	3.088156239	0.025347319	Streptomycin biosynthesis	Biosynthesis of other secondary metabolites	Metabolism
**ko00525**	Vehicle	2.750138003	0.025347319	Acarbose and validamycin biosynthesis	Biosynthesis of other secondary metabolites	Metabolism
**ko00940**	Vehicle	3.384682193	0.025347319	Phenylpropanoid biosynthesis	Biosynthesis of other secondary metabolites	Metabolism
**ko00944**	Vehicle	2.124116539	0.025347319	Flavone and flavonol biosynthesis	Biosynthesis of other secondary metabolites	Metabolism
**ko00950**	Vehicle	2.512293844	0.025347319	Isoquinoline alkaloid biosynthesis	Biosynthesis of other secondary metabolites	Metabolism
**ko00966**	Vehicle	2.559374797	0.025347319	Glucosinolate biosynthesis	Biosynthesis of other secondary metabolites	Metabolism
**ko00998**	Vehicle	3.597845896	0.025347319	Biosynthesis of various secondary metabolites - part 2	Biosynthesis of other secondary metabolites	Metabolism
**ko00195**	Vehicle	2.858501721	0.025347319	Photosynthesis	Energy metabolism	Metabolism
**ko00680**	Vehicle	2.88028891	0.025347319	Methane metabolism	Energy metabolism	Metabolism
**ko00710**	Vehicle	3.650154562	0.025347319	Carbon fixation in photosynthetic organisms	Energy metabolism	Metabolism
**ko00720**	Vehicle	3.136399772	0.025347319	Carbon fixation pathways in prokaryotes	Energy metabolism	Metabolism
**ko00621**	Vehicle	2.814246347	0.025347319	Dioxin degradation	Xenobiotics biodegradation and metabolism	Metabolism
**ko00622**	Vehicle	2.815249564	0.025347319	Xylene degradation	Xenobiotics biodegradation and metabolism	Metabolism
**ko00791**	Vehicle	2.841941817	0.025347319	Atrazine degradation	Xenobiotics biodegradation and metabolism	Metabolism
**ko00983**	Vehicle	2.635073652	0.025347319	Drug metabolism - other enzymes	Xenobiotics biodegradation and metabolism	Metabolism
**ko00523**	Vehicle	2.944006041	0.025347319	Polyketide sugar unit biosynthesis	Metabolism of terpenoids and polyketides	Metabolism
**ko00908**	Vehicle	2.200723171	0.025347319	Zeatin biosynthesis	Metabolism of terpenoids and polyketides	Metabolism
**ko00361**	Antibiotic	2.922657228	0.021946771	Chlorocyclohexane and chlorobenzene degradation	Xenobiotics biodegradation and metabolism	Metabolism
**ko00362**	Antibiotic	3.124524256	0.025347319	Benzoate degradation	Xenobiotics biodegradation and metabolism	Metabolism
**ko00364**	Antibiotic	2.922654656	0.021946771	Fluorobenzoate degradation	Xenobiotics biodegradation and metabolism	Metabolism
**ko00625**	Antibiotic	2.27660938	0.025347319	Chloroalkane and chloroalkene degradation	Xenobiotics biodegradation and metabolism	Metabolism
**ko00624**	Antibiotic	2.727709127	0.021946771	Polycyclic aromatic hydrocarbon degradation	Xenobiotics biodegradation and metabolism	Metabolism
**ko00627**	Antibiotic	2.917972825	0.025347319	Aminobenzoate degradation	Xenobiotics biodegradation and metabolism	Metabolism
**ko00626**	Antibiotic	2.290292359	0.025347319	Naphthalene degradation	Xenobiotics biodegradation and metabolism	Metabolism
**ko00623**	Antibiotic	2.922598893	0.021946771	Toluene degradation	Xenobiotics biodegradation and metabolism	Metabolism
**ko00633**	Antibiotic	2.80222781	0.025347319	Nitrotoluene degradation	Xenobiotics biodegradation and metabolism	Metabolism
**ko00642**	Antibiotic	2.833639641	0.021946771	Ethylbenzene degradation	Xenobiotics biodegradation and metabolism	Metabolism
**ko00930**	Antibiotic	2.827951192	0.021946771	Caprolactam degradation	Xenobiotics biodegradation and metabolism	Metabolism
**ko00900**	Antibiotic	2.645723217	0.025347319	Terpenoid backbone biosynthesis	Metabolism of terpenoids and polyketides	Metabolism
**ko00903**	Antibiotic	2.717177341	0.024480834	Limonene and pinene degradation	Metabolism of terpenoids and polyketides	Metabolism
**ko01051**	Antibiotic	2.404187819	0.025347319	Biosynthesis of ansamycins	Metabolism of terpenoids and polyketides	Metabolism
**ko01053**	Antibiotic	2.250155751	0.025347319	Biosynthesis of siderophore group nonribosomal peptides	Metabolism of terpenoids and polyketides	Metabolism
**ko00281**	Antibiotic	2.97682536	0.025347319	Geraniol degradation	Metabolism of terpenoids and polyketides	Metabolism
**ko00190**	Antibiotic	3.24908232	0.025347319	Oxidative phosphorylation	Energy metabolism	Metabolism
**ko00920**	Antibiotic	3.649946377	0.025347319	Sulfur metabolism	Energy metabolism	Metabolism
**ko00230**	Antibiotic	2.910427752	0.025347319	Purine metabolism	Nucleotide metabolism	Metabolism
**ko00524**	Antibiotic	2.039274476	0.025347319	Neomycin, kanamycin and gentamicin biosynthesis	Biosynthesis of other secondary metabolites	Metabolism
