# Supplementary material for: Antibiotic-Induced Primary Biles Inhibit SARS-CoV-2 Endoribonuclease Nsp15 Activity in Mouse Gut
Source: Front Cell Infect Microbiol. 2022 Jul 28;12:896504. doi: 10.3389/fcimb.2022.896504 (PMC9366059; doi:10.3389/fcimb.2022.896504)
Supplement: Table S3 — Annotation of gene families related to bile acid metabolism. [file DataSheet_8.pdf]

Table S3. Annotation of gene families related to bile acid metabolism.

**Relative abundance of enriched functional genes in mouse fecal metagenomes.**							
		Function					
SampleID	Group	K22605	K00076	K15873	K01442	Secondary bile acid biosynthesis	Primary bile acid biosynthesis
A13	Antibiotic	0	0	0	0	0	0
A14	Antibiotic	0	1.64E-05	0	0	1.64E-05	0
A15	Antibiotic	0	0	0	0	0	0
A16	Antibiotic	0	3.40E-05	0	0	3.40E-05	0
A17	Antibiotic	0	9.40E-06	0	0	9.40E-06	0
A31	Vehicle	0.00163679	0	0	0.0479765	0.04961329	0.0479765
A32	Vehicle	0	0	0.00263614	0.0162657	0.01890184	0.0162657
A33	Vehicle	0	0.0027659	0	0.0282003	0.0309662	0.0282003

**Statistics of Wilcoxon test for identifying the differential features between the antibiotic and vehicle groups.**	
Function	*p* -value
K22605	0.3017
K00076	1
K15873	0.3017
K01442	0.0168
Secondary bile acid biosynthesis	0.0358
Primary bile acid biosynthesis	0.0168
